# Predictors of Out of Hospital Cardiac Arrest Outcomes in Pre-Hospital Settings; a Retrospective Cross-sectional Study

**Published:** 2019-07-10

**Authors:** Elham Navab, Maryam Esmaeili, Nastaran Poorkhorshidi, Rasoul Salimi, Afshin Khazaei, Abbas Moghimbeigi

**Affiliations:** 1 Critical Care and Geriatric Nursing Department, School of Nursing and Midwifery, Tehran University of Medical Sciences, Tehran, Iran.; 2 Student Research Committee, Faculty of Nursing and Midwifery, Shahid Beheshti University of Medical Sciences, Tehran, Iran.; 3 Emergency Department, Besat Hospital, Hamedan University of Medical Sciences, Hamedan, Iran.; 4 Intensive Care and Management Nursing Department, School of Nursing and Midwifery, Hamedan University of Medical Sciences, Hamedan, Iran.; 5 Professor of Epidemiology and Biostatistics, Department of Biostatistics and Epidemiology, School of Public Health, Hamedan University of Medical Sciences, Hamedan, Iran.

**Keywords:** Out-of-Hospital Cardiac Arrest, Emergency Medical Services, Emergency Medical Technicians, Cardiopulmonary Resuscitation

## Abstract

**Introduction:**

Different potential factors can affect the outcomes of Out of Hospital Cardiac Arrest (OHCA). The present study aimed to identify important factors contributing to the Return of Spontaneous Circulation (ROSC) and Survival to Hospital Discharge (SHD) in these patients.

**Methods:**

This cross-sectional study was conducted on all the OHCA patients who underwent Cardiopulmonary Resuscitation (CPR) in emergency medical service (EMS) of Hamedan province during 2016-2017. All the relevant data were retrieved from three sources, according to Utstein’s style. In addition, univariate and multivariate logistic regressions were employed to identify predictive factors of ROSC and SHD using SPSS software, version 20.

**Results:**

Among the 3214 eligible patients whose data were collected, most OHCA patients were female (59.7%) with the mean age of 58 years. Moreover, the majority of OHCAs (77.8%) occurred at home during 8pm-8am (65.1%) and about 26.3% of OHCAs were witnessed, with only 5.1% bystander-initiated CPR. Furthermore, the median ambulance response time and CPR duration were 6.0 and 20 minutes, respectively. Overall, ROSC and SHD success rates were 8.3 and 4.1%, respectively. Bystander CPR was found to be the most effective predicting factor for the success rate of ROSC (AOR=3.26, P<0.001) and SHD (AOR=3.04, P<0.001) after adjusting for the Utstein variables including the patients’ age, gender, cardiac disease history, arrest time, CPR duration, response time, being witnessed, bystander CPR, and endotracheal intubation (ETI).

**Conclusion:**

The overall success rates of ROSC and SHD were 8.3% and 4.1%, respectively. The age, ambulance response time, CPR duration, and cardiac disease history were negatively associated with the outcomes of ROSC and SHD, while being witnessed, bystander CPR, ETI, and initial shockable rhythm were positively related to both of the above-mentioned outcomes.

## Introduction

Out of hospital cardiac arrest (OHCA) is considered as one of the leading causes of mortality around the world due to its high incidence, low survival rate, and unpredictability, and has attracted much attention in recent years (1). The incidence of OHCAs was reported to be an average of 84 events per each 100,000 population in 27 European countries (2). More than 575,000 OHCA cases occur annually in North America (3) and its social and clinical impact is so high that, the American Heart Association (AHA) has recommended reporting the outcomes of OHCAs (4). For this purpose, the Clinical Research Network established the Pan-Asian Resuscitation Outcomes Study in seven Asian countries in 2010 (5). This network also focuses on improving the outcomes of Prehospital and emergency care across the Asia-Pacific region by performing high-quality research in cardiopulmonary resuscitation (CPR).

Management and treatment of OHCAs are regarded as the main challenges in the Emergency Medical Services (EMS), considering the demanding nature of OHCA (1). Furthermore, CPR is considered as one of the most important measures in OHCAs, which is a vital and effective procedure for determining the patient’s final outcome (1). Although resuscitation knowledge has dramatically increased over the past few decades, no significant improvement is observed regarding the rate of return of spontaneous circulation (ROSC) and survival to hospital discharge (SHD) among OHCA patients (6, 7). The latest CPR protocol (2015) has been recommended by the AHA concerning guidance on OHCA care including basic life support (BLS) and advanced life support (ALS) care (8). These interventions are performed by EMS in most countries (almost 60%) with substantial variations in CPR outcome (9). 

Accordingly, access to basic, accurate, and reliable data related to OHCA outcomes, and their related underlying factors such as the characteristics of patients and OHCA are considered potentially effective factors in this regard. In other words, it provides opportunities for the researchers and policymakers, as well as those seeking to collect OHCA-related data to use appropriate scientific approaches for prioritizing the resources, monitoring the national trends in OHCA survival, reducing OHCA-related costs, promoting high-quality research, and improving OHCA care (3, 10). Therefore, due to scarcity of data in this regard, the present study primarily sought to determine the success rate of ROSC and SHD and identify the most influential factors.

## Methods

 ***Study Design and Setting***

The current multicenter cross-sectional study was conducted in the emergency medical service center of Hamedan province (located in the west of Iran), which included 20 urban and 30 road bases with the population of about two million people, during (April) 2016-(February) 2017. In this setting, all of the steps of CPR were performed based on the American Heart Association Guidelines for CPR (2015), which required any deployed technician in OHCA-related missions to provide basic and advanced life support care according to the guidelines. The present research was approved by Ethics Committee of Hamedan University of Medical Sciences (No: IR.UMSHA.REC.1396.808).

***Participants***

All data of OHCA patients treated by the Emergency Medical Technicians (EMTs) at urban bases were collected retrospectively. The inclusion criteria consisted of OHCA patients aged ≥18 years and the presumed cardiac etiology transported to the hospital by the EMTs. The exclusion criteria encompassed a non-cardiac cause of OHCA, incomplete documents and cases with missing data, EMTs witnessing OHCA, patients who were obviously dead at the scene (i.e., rigor mortis, lividity, decomposition, or decapitation) with no attempt at CPR by the EMTs, and those who achieved ROSC or available cardiac support at the scene before the arrival of the EMTs.

***Data gathering***

The 2015 Utstein Resuscitation Registry style was employed for recording OHCA reports (11). All data were obtained from three sources including the Registration System of Information and Statistics, Patient Care Report form, and Hospital Information System. Patients were excluded if cardiac arrest occurred in a clinic or where other emergency providers were attending to the patient. The data extracted for analysis included patients’ characteristics such as gender and age, as well as a history of cardiac diseases and OHCA characteristics including the time of arrest (i.e., 8am-8pm/8pm-8am), event being witnessed, the provision of bystander-initiated CPR, the location of cardiac arrest (home/public), ambulance response time (defined by the time interval between a call and CPR initiation), initial shockable rhythm, CPR duration, endotracheal intubation (ETI), and outcome (ROSC and SHD). 

***Statistical Analysis***

Continuous variables were expressed as mean ± standard deviation (SD) or median and interquartile ranges (IQRs) where appropriate. Furthermore, categorical variables were demonstrated as frequency and percentage. Additionally, univariate (in each group) and multivariate (for the adjusted model) logistic regressions were applied to identify ROSC and SHD predictive factors through an association between continuous and categorical variables. The potential confounding variables in the final adjusted model were selected by a forward selection (Wald) method (i.e., entering [0.005] and removing [0.10] predictors). In addition, Utstein (a set of guidelines for uniform reporting of cardiac arrest) factors were utilized to select all the covariates that were recommended for reporting OHCA-related data (12). The area under the receiver operating characteristic (ROC) curve of the final model for predicting the probability of ROSC and SHD were calculated. The odds ratios with 95% *confidence intervals (CI)* were calculated as well. Eventually, all the statistical analyses were performed using the IBM SPSS Statistics software, version 20. 

**Figure 1:**
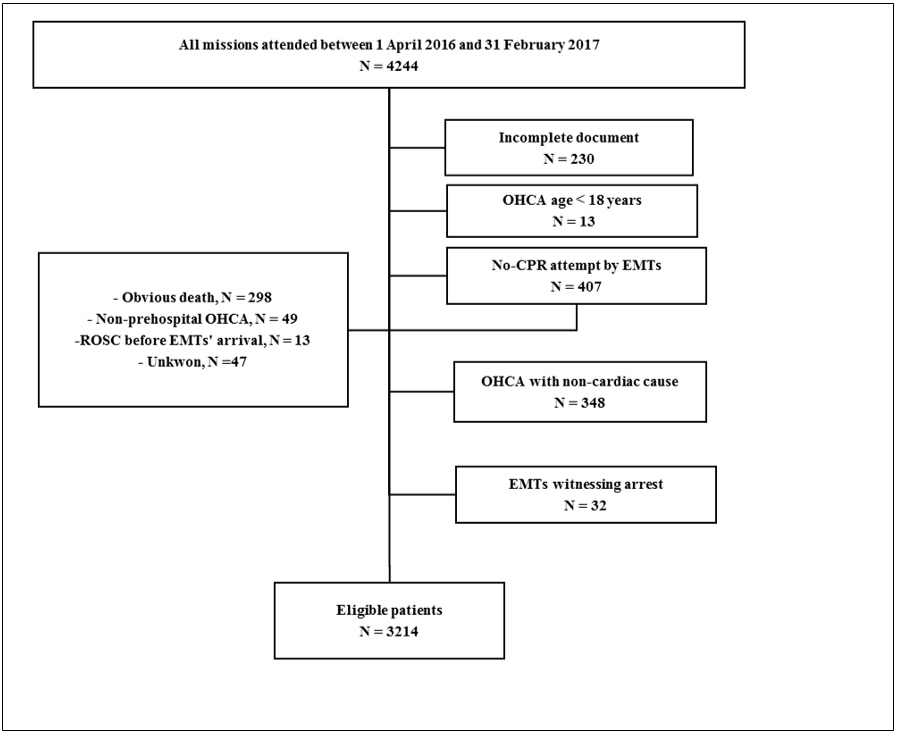
Flowchart of patient enrollment. ROSC: return of spontaneous circulation; EMTs: emergency medical technicians, OHCA: out-of-hospital cardiac arrest

**Table 1:** Comparing the baseline characteristics of out of hospital cardiac arrest cases based on the return of spontaneous circulation (ROSC) and survival to hospital discharge (SHD)

**Variable**	**ROSC**		**SHD **	
	No (n=2941)	Yes (n=273)	No (n=3088)	Yes (n=126)
**Age (year)**				
Mean ± SD	58.1 ± 11.7	54.9 ± 14.8	57.8 ± 13.1	54.9 ± 12.0
**Gender**				
Female	1747 (91.9)	153 (8.1)	1817 (95.6)	83 (4.4)
Male	1201 (91.4)	113 (8.6)	1266 (96.3)	48 (3.7)
**Cardiac history**				
Yes	1957 (93.7)	132 (6.3)	2018 (96.6)	71 (3.4)
No	991 (81.18)	134 (11.9)	1065 (94.7)	60 (5.3)
**Witnessed arrest**				
Yes	746 (88.2)	100 (11.8)	512 (95.5)	24 (4.5)
No	2195 (92.7)	173 (7.3)	2300 (97.1)	68 (2.9)
**CPR by bystander**				
Yes	129 (72.1)	34 (29.0)	146 (89.6)	17 (10.4)
No	2819 (92.4)	232 (7.6)	2937 (96.3)	114 (3.5)
**Shockable r** **hythm**				
Yes	651 (85.9)	107 (14.1)	707 (93.3)	51 (6.7)
No	2297 (93.5)	159 (6.5)	2276 (96.7)	80 (3.3)
**Arrest location**				
Home	2112 (92.8)	163 (7.2)	2194 (96.4)	81 (3.6)
Public	836 (89.0)	103 (11.0)	889 (94.7)	50 (5.3)
**Response time (minutes)**				
Mean ± SD	6.81 ± 3.35	4.73 ± 2.65	6.74 ± 3.32	4.27 ± 3.03
**CPR duration ** **(minutes)**				
Mean ± SD	22.4 ± 12.88	16.9 ± 10.40	23.1 ± 12.91	17.1 ± 8.35
**Arrest time**				
Day	962 (92.3)	80 (7.7)	1011 (97)	31 (3.0
Night	1986 (91.4)	186 (8.6)	2072 (95.4)	100 (4.6)
**ETI **				
Yes	613 (88.6)	79 (11.4)	658 (95.1)	34 (4.9)
No	2235 (92.6)	187 (7.4)	2425 (96.2)	97 (3.8)

Data are presented as mean ± standard deviation (SD) or number (%). CPR: Cardiopulmonary Resuscitation; ETI: Endotracheal Intubation. CPR duration: was defined as the time from initiation of CPR by emergency medical technicians to prehospital return of spontaneous circulation.

**Table 2:** Effective factors of return of spontaneous circulation (ROSC) in out-of-hospital cardiac arrest cases based on univariate and multivariate regression analyses

**Variable (reference) **	**Univariate**		**Multivarate**	
	**OR (95% CI)**	**P value**	**AOR (95% CI)**	**P value**
**Patient age**	0.96 (0.95-0.97)	< 0.001	0.97 (0.95-0.98)	< 0.001
**Gender (male)**	1.07 (0.83-1.38)	0.580	-	-
**Arrest time (night)**	0.88 (0.67-1.16)	0.394	-	-
**Arrest location (home)**	1.59 (1.23-2.06)	< 0.001	1.47 (1.94 -1.11)	0.007
**CPR duration**	0.95 (0.94-0.96)	< 0.001	0.95 (0.93-0.96)	< 0.001
**History of cardiac disease (No)**	0.49 (0.38-0.64)	< 0.001	0.54 (0.40 – 0.72)	< 0.001
**Witnessed (No)**	1.46 (1.12-1.90)	0.005	-	-
**Bystander CPR (No)**	3.20 (2.14-4.78)	< 0.001	3.26 (2.08 – 5.12)	< 0.001
**Response time **	0.79 (0.75-0.83)	< 0.001	0.80 (0.76-0.84)	< 0.001
**ETI (No)**	1.60 (1.21-2.12)	0.001	1.63 (1.21 – 2.20)	0.001
**Shockable rhythm (No)**	2.37 (1.83-3.08)	< 0.001	1.86 (1.41 -2.46)	0.001

CI: confidence interval; CPR: cardiopulmonary resuscitation; ETI: endotracheal intubation; OR: Odds ratio; AOR: Adjusted Odds ratio (adjusted for patients’ age, gender, arrest location, history of cardiac disease, time of arrest, CPR duration, response time, witnessed, bystander CPR and endotracheal intubation and shockable rhythm).

**Table 3:** Effective factors of survival to hospital discharge (SHD) in out-of-hospital cardiac arrest cases based on univariate and multivariate regression analyses

**Variable (reference)**	**Univariate**		**Multivarate**	
	**OR (95% CI)**	**P value**	**AOR (95% CI)**	**P value**
**Patient age**	0.98 (0.96-0.99)	0.010	0.98 (0.96-0.99)	0.011
**Gender (male)**	0.83 (0.57-1.19)	0.314	-	-
**Arrest Time (night)**	0.63 (0.42-0.95)	0.030	-	-
**Arrest location (home)**	1.52 (1.06-2.18)	0.022	-	-
**CPR duration**	0.73 (0.68-0.79)	< 0.001	0.96 (0.94-0.97)	< 0.001
**History of cardiac disease (No)**	0.62 (0.43-0.88)	0.009	0.59 (0.41-0.85)	0.006
**Witnessed (No)**	1.86 (1.30-2.66)	0.001	-	-
**Bystander CPR (No)**	3.00 (1.12-5.12)	< 0.001	3.04 (1.73-5.35)	< 0.001
**Response Time**	0.83 (0.78-0.89)	< 0.001	0.74 (0.69-0.80)	< 0.001
**ETI (No)**	1.29 (0.86-1.92)	< 0.001	-	-
**Shockable Rhythm (No)**	2.14 (1.49-3.07)	< 0.001	1.79 (1.23-2.6)	0.002

CI: confidence interval; CPR: cardiopulmonary resuscitation; ETI: endotracheal intubation; OR: Odds ratio; AOR: Adjusted Odds ratio (adjusted for patients’ age, gender, arrest location, history of cardiac disease, time of arrest, CPR duration, response time, witnessed, bystander CPR and endotracheal intubation and shockable rhythm).

**Figure 2:**
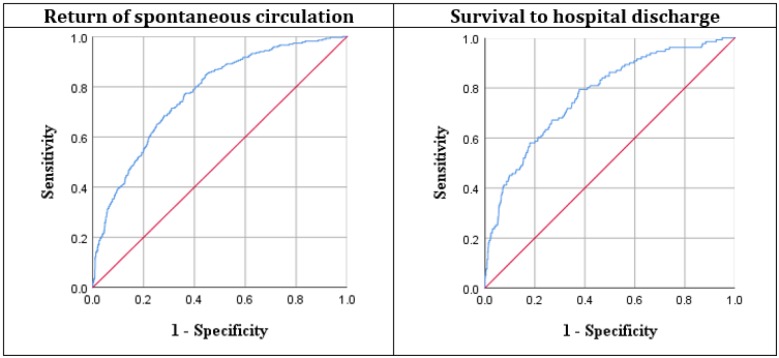
Area under the receiver operating characteristic (ROC) curve of adjusted logistic regression model for predicting the return of spontaneous circulation (0.771 (95% CI: 0743 – 0.799)) and survival to hospital discharge (0.772 (95% CI: 0.731– 0.813))

## Results

***Baseline characteristics of studied cases***

4244 OHCA reports were evaluated, among which 1030 were excluded (Figure 1). Finally, 3214 patients with the mean age of 58.47 ± 12.10 years were included in the final analysis (59.7% female). The rate of SHD was higher in females (4.4% vs. 3.7%; p = 0.313) while the rate of ROSC was higher in males (8.1% vs. 8.6%; p = 0.580). Most OHCAs occurred at home (77.8%) and during 8pm-8am (65.1%). 26.3% of the OHCAs were witnessed with a total of 5.1% bystander-initiated CPR and most of the witnessed OHCAs were observed in the public, compared to home (2.8% vs. 1.7%). The median ambulance response time and CPR duration were 6.0 (IQR: 4-9) and 20 (IQR: 12-33) minutes, respectively. Regarding the initial arrest rhythm, the cases had mainly shown non-shockable (76.4%) rhythms, while most of the shockable rhythms were found in patients with a history of heart disease (13.8% vs. 9.8%). The number of successful ROSC (16.9% vs. 4.7%) and SHD (7.0% vs. 2.6%) cases was higher in patients with no history of heart disease and initial shockable rhythm. The rate of out-of-hospital ETI placement was only 21.4%. In general, the success rates of ROSC and SHD were 8.3% and 4.1%, respectively. Additional details of the patients’ characteristics are presented in table 1. 

***Results of Univariate and Multivariate Analysis***

Tables 2 and 3 represent the univariate and multivariate logistic regression analyses of ROSC and SHD effective factors. Based on unadjusted regression, bystander CPR and initial shockable rhythm were the most important factors influencing ROSC and SHD, respectively. Bystander CPR was the most effective predicting factor for ROSC (AOR: 3.26, P<0.001) and SHD (AOR: 3.04, P<0.001) in OHCA patients after adjusting for nine variables including the patients’ age, gender, cardiac disease history, the time of arrest, CPR duration, response time, being witnessed, bystander CPR, and endotracheal intubation (ETI).

 ROC curve of the final model is presented in figure 2. The specificity and positive predictive value of the predicting model for SHD were 77.1% (95% CI: 74.3-79.9) and 77.2% (95% CI: 73.1-81.3), respectively. 

## Discussion

 Based on the results of the present study, the age, ambulance response time, CPR duration, and cardiac disease history were negatively associated with the outcomes of ROSC and SHD while being witnessed, bystander CPR, ETI, and initial shockable rhythm were positively related to both of the above-mentioned outcomes. 

The overall success rates of ROSC and SHD were 8.3% and 4.1%, respectively, among the patients who underwent CPR by EMTs. The rate is low compared to that of the other studies in this area. Based on the reports, the results of OHCA registries regarding SHD rate ranged from 7.5% to 10.8% in the United States and Europe (13, 14). Conversely, this rate was found to be only 5.4% of OHCA patients in the Pan-Asian Resuscitation Outcomes Study registry (15) and a meta-analysis achieved a pooled SHD rate of 7.6% (12).

Based on the findings of previous studies (16, 17), age is regarded as the predictor of OHCA outcome. The SHD rate of the elderly OHCA patients compared to younger patients was approximately half (18). In the present study, the mean ages of the patients with ROSC and SHD, were nearly 4 and 3 years less than that of the other patients, respectively. In addition, the unadjusted analysis revealed that each year increment in the age decreased the probability of ROSC and SHD by 1.03% and 1.04%, respectively. Furthermore, in the adjusted regression analysis, age still had a significant inverse association with ROSC (OR: 0.97, P<0.001) and SHD (OR: 0.98, P=0.011), the details of which are provided in Tables 2 and 3. 

Witnessed cardiac arrest with CPR initiation by bystanders, preferably before the arrival of EMTs, is regarded as one of the important predictive factors in OHCA outcome, which can increase SHD about 40% and improves the neurological symptoms of the patients (19, 20). More importantly, timely bystander CPR can improve the outcomes despite the prolonged on-scene times by EMTs (21). In some advanced countries, CPR is increasingly performed by bystanders, the rates of which have reached 50% (13). However, bystander CPR rate was very low in both witnessed (2.0%) and non-witnessed (3.1%) cases in the current study. The result becomes more prominent when CPR by bystanders was found to increase the chances of successful CPR and SHD to 1.50 (OR: 3.89 vs. 2.59, P=0.001) and 1.35 fold (OR: 3.16 vs. 2.33, P<0.001) in the unadjusted regression, compared to the non-witnessed. The results are consistent with those of other studies regarding OHCAs. Rajan et al. indicated that quick and uninterrupted CPR by bystanders before the arrival of the ambulance at the scene can lead to more than two-fold increase in the one-month survival rate of the patients (22). 

Initial shockable rhythm (i.e., Ventricular Tachycardia) is another essential factor in OHCA, and the success rate of ROSC and SHD dramatically increases in case of timely shock using defibrillator (23). Based on the results of the present study concerning the unadjusted regression, patients with an initial shockable rhythm shocked by the EMT had a higher chance of CPR success (OR: 2.37, P<0.001) and SHD rate (OR: 2.14, P<0.001) compared to those with a non-shockable rhythm. When adjusted for other variables, the initial shockable rhythm was still an important effective factor on the outcomes of CPR (AOR: 1.86, P=0.001) and SHD (AOR: 1.79, P=0.002) compared to the non-shockable rhythm (Tables 2 and 3). In this regard, Public Access Defibrillation and the related programs such as leadership behavior, training, competency, and experience in managing emergencies (24), especially in the areas with a higher incidence rate of OHCA can be effective in CPR success and improving the survival rate of the victims (25). Furthermore, Stammet et al. found that OHCA patients who underwent CPR by a bystander and those who used automated external defibrillators (AED) had almost 1.2 and 2.28 times better CPR outcomes, respectively, compared to those who underwent CPR without the AED and the patients with non-witnessed OHCA (26). Additionally, the results of a systematic review and meta-analysis demonstrated that survival chance and favorable neurological status were nearly doubled when the AED device was used by a bystander during the CPR (20). AED device is widely used in cardiac arrests in public places since almost 20% of the OHCA cases occur in such places (27). As shown in Table 2, the highest success rates regarding CPR (11.0% vs. 7.2%) and SHD (5.3% vs. 3.6%) in OHCAs belong to public places due to higher probability of the arrest being witnessed. Unfortunately, no AED devices existed in public places of Hamedan province, leading to an increase in the number of OHCA patients. Given the findings of evidence-based studies, the American Heart Association and the European Resuscitation Council recommended to implement programs related to quick access to the defibrillator device in their 2015 guidelines (28, 29). Therefore, the necessity of installing AED devices in public places and implementing the general CPR training program at the community level are considered factors that can play a key role in OHCA outcomes in the present context. 

In addition, the time of arrest (night/day), as well as its effect on the outcomes of OHCA was investigated in the current study. The result of univariate regression indicated that the time of arrest only affected SHD (OR: 0.63, P=0.030), while it no longer showed an effect on SHD in the multivariate regression and the difference in survival of the subgroups disappeared when adjusted for the confounders. In line with the result of the present study, in a meta-analysis study, Lin et al. concluded that patients who had an OHCA during the night had lower 1-month/in-hospital survival, compared to those with daytime OHCA (30). Additionally, Ho AFW et al. (31) reported that the 30-day survival of OHCA cases happening at night was lower than those happening in the daytime among the Pan-Asians with an adjusted odds ratio of 0.79. Perhaps, one of the reasons for the effect of the arrest time on CPR outcomes in the current study is that OHCA at night is less likely to be witnessed (7.7 vs. 20.1%, P<0.001) and receive bystander CPR (2.0 vs. 3.1%, P=0.05).

Furthermore, the duration of prehospital CPR before transporting the patients to a hospital is regarded as another key component in OHCA outcomes (12). Despite the major advances in CPR, no comprehensive agreement was reached with respect to the duration of CPR in OHCA and acceptable time for its termination. Based on the results of the present study, the mean duration of CPR in the successful ROSC was less than the unsuccessful ROSC, and the SHD versus non-SHD. According to previous reports, Reynolds et al. (32) determined that ROSC occurs in 89.7% of OHCA patients who undergo CPR within 16 minutes. Additionally, Cooper et al. reported that the CPR duration of less than 14 minutes caused a 62% survival rate among OHCA patients while it was 20% for CPR durations over 15 minutes (33). In addition, according to Funada et al., CPR duration over 26 minutes led to ROSC failure in OHCA patients (34). Furthermore, the results of both adjusted and unadjusted regression analyses of the current study demonstrated that CPR duration was independently and inversely associated with successful ROSC and SHD (Tables 2 and 3). After confounder adjustment, every minute increase in CPR duration was found to be related to a 9% reduction in the odds of successful CPR (AOR 0.95, CI: 0.93-0.96, P<0.001) and SHD (AOR 0.95, CI: 0.94-0.97, P<0.001). Dyson et al. obtained the same result by assessing the impact of CPR duration on SHD in their study (35). Therefore, the lack of an appropriate termination of resuscitation rule in BLS and ALS care can lead to an increase in patient transport to the hospital, ineffective attempts, a waste of medical resources, and the exposure of tired EMT and the public at the risk of accident due to the high-speed transport (36). However, there are still challenges that remain unsolved regarding appropriate determination of resuscitation termination for OHCA patients (37).

The survival rate of cardiac arrest decreases 5-10% for every minute that passes from the event (38). In this regard, the response time of the emergency medical service is regarded as one of the important factors associated with ROSC and SHD in OHCA cases (39). In the current study, the mean ambulance response time in unsuccessful ROSC was 1.43 times more than the successful ROSC. Furthermore, this comparison in non-SHD and SHD was 1.57 times, which confirmed a significant inverse relationship between ambulance response time and the success/failure of ROSC and SHD. Additionally, the results of multivariate logistic regression test indicated that the success rates of ROSC and SHD dropped 1.24 and 1.33 times, respectively, for each minute of delay in initiation of CPR. A large body of research reported different results regarding the effect of the response time on CPR outcomes. For example, Sladjana et al. found that higher SHD and ROSC rate is observed when CPR is performed within the first four minutes after the OHCA (40). Burger et al. demonstrated that if CPR is performed by a bystander and the mean ambulance response time increases from 1:04 to 9:47 minutes, the discharge rate reduces from 22.0% to 14.0%; while if no bystander CPR is performed and the mean ambulance response time increases from 1:10 to 9:47 minutes, the discharge rate drops from 12.9% to 6.4% (41). Therefore, based on the findings, identifying the areas and geographic locations with higher incidence of OHCAs can help reduce the response time and improve the SHD rate (40). In this regard, emergency operators can play an important role by using telephone CPR, training bystanders, and increasing the number of bystander CPR rate for improving favorable outcomes (42). 

Finally, the effect of prehospital Advanced Airway Management (AAM) in OHCA patients is still considered as a controversial issue (43). ETI is regarded as the optimal method and the gold standard of AAM in the prehospital setting (44, 45). Performing it can be associated with improved odds of sustained ROSC, SHD, and favorable neurologic outcomes (43). Based on the results of univariate regression, ETI in OHCA was an effective factor in the success of ROSC (OR:1.60, P=0.001) and SHD (OR:1.29, P<0.001), and this factor only correlated with ROSC in the presence of other covariates in the adjusted model (OR:1.63, P=0.001). Benoit et al. (46) concluded that delay in ETI was associated with decreased probability of ROSC, which is in conformity with the results of the current study. In addition, Izawa et al. demonstrated that a shockable rhythm determines the effect of ETI placement on OHCA and performing AAM in patients with non-shockable rhythm showed better survival compared to those with a shockable rhythm (47). However, evaluating the effect of this factor on OHCA outcomes is vital, which necessitates further investigation.

In the end, since the study was conducted for patients over the age of 18 years, the results cannot be interpreted for those under the age of 18. Also, 7.1% (230) of the information of patients who have suffered from OHCA have been excluded due to incomplete information, most of which was related to the bystander-initiated CPR and initial shockable rhythm; therefore, the results of this study should be used with caution. The results of the present study revealed that the rate of success in ROSC and SHD in the Emergency Medical Services of Hamedan province was extremely low compared to those of previous studies. Accordingly, the results of this study showed us that some modifiable predictive factors could improve the ROSC and SHD rate in a limited-resource setting such as the prehospital emergency. Therefore, the two above-mentioned outcomes can be applied to enhance ROSC and SHD rates by improving the important modifiable contributing factors such as provision of bystander-initiated CPR, ETI, and reducing ambulance response time.

## Limitations

One limitation of the current study was its retrospective design, so that the accuracy of data collection could not be monitored and there could be potential measured and unmeasured confounders, which may account for the observed outcomes. In the present study, the lack of an integrated system for recording the data of the patients who suffered from OHCA after one month led to the impossibility of assessing two important factors, namely, neurological status and patient survival one month after the cardiac arrest and its association with CPR outcomes. Furthermore, the lack of access to the AED device by the bystanders led to the impossibility of measuring the effect of using the defibrillator on the outcomes of CPR performed by the bystanders on OHCA patients.

## Conclusion:

 Based on the results of the present study, age, ambulance response time, CPR duration, and cardiac disease history were negatively associated with ROSC and SHD outcomes, while being witnessed, bystander CPR, ETI, and initial shockable rhythm were positively related to both of the above-mentioned outcomes. The overall success rates of ROSC and SHD were 8.3% and 4.1%, respectively.
